# A Dictionary of Practical Medicine, Comprising General Pathology, and the Nature and Treatment of Diseases, Morbid Structures, &c.

**Published:** 1844-12

**Authors:** 


Art. VIII.—*A Dictionary of Practical Medicine*, comprising General Pathology, and the nature and treatment of Diseases, morbid structures, &c. By James Copland, M.D.F. R.S. Edited, with additions. By Charles A. Lee, M.D., New York: Henry G. Langley. 1844. 8vo. Part I. p.p. 144.

At length the American profession is favored with an opportunity of procuring, on easy terms, this most admirable dictionary of practical medicine, the fame of which has preceded its appearance, and spreac far and wide over the country. Few works have met with as much public favor at home; few, indeed, have received more labor from the author, or comprise in as small space so great an amount of valuable practical matter. All we deem necessary is to publish the terms on which the work is to be issued, for no new commendation is required to secure for it an extensive sale. It is to be published in twenty monthly numbers, making three large octavo volumes, at ten dollars. This Dictionary will constitute, when completed, a library of great value to the practitioner.

